# A systematic appraisal of allegiance effect in randomized controlled trials of psychotherapy

**DOI:** 10.1186/s12991-015-0063-1

**Published:** 2015-09-15

**Authors:** Elena Dragioti, Ioannis Dimoliatis, Konstantinos N. Fountoulakis, Evangelos Evangelou

**Affiliations:** Department of Hygiene and Epidemiology, University of Ioannina Medical School, Ioannina, Greece; Division of Community Medicine, Department of Medical and Health Sciences, Faculty of Health Sciences, Linköping University, Pain and Rehabilitation Center, Anesthetics, Operations and Specialty Surgery Center, County Council of Östergötland, 58185 Linköping, Sweden; 3rd Department of Psychiatry, School of Medicine, Aristotle University of Thessaloniki, Thessaloniki, Greece; Department of Biostatistics and Epidemiology, School of Public Health, Imperial College London, London, UK

**Keywords:** Allegiance effect, Experimenter’s allegiance, Psychotherapy, Optimism bias, Non-financial conflict of interest, Systematic bias

## Abstract

**Background:**

Experimenter’s allegiance (EA) refers to a personal confidence of the superiority of a specific psychotherapy treatment. This factor has been linked with larger treatment effects in favor of the preferred treatment. However, various studies have displayed contradictory results between EA and the pattern of treatment effects.

**Aims:**

Using a systematic approach followed by meta-analysis, we aimed to evaluate the impact of an allegiance effect on the results of psychotherapeutic studies.

**Method:**

We considered the meta-analyses of randomized controlled trials (RCTs) of different types of psychotherapies in the Cochrane Database of Systematic Reviews. Eligible articles included meta-analyses of RCTs with at least one study showing evidence of EA (i.e., allegiant study). Effect sizes in allegiant RCTs were compared with non-allegiant using random and fixed models and a summary relative odds ratio (ROR) were calculated. Heterogeneity was quantified with the *I*^2^ metric.

**Results:**

A total of 30 meta-analyses including 240 RCTs were analyzed. The summary ROR was 1.31 [(95 % confidence interval (CI: 1.03–1.66) *P* = 0.30, *I*^2^ = 53 %] indicating larger effects when allegiance exists. The impact of allegiance did not differ significantly (*P* > 0.05) when we compared psychiatric versus medical outcomes. Allegiance effect was significant for all forms of psychotherapy except for cognitive behavioral therapy. Moreover, the impact of allegiance was significant only when the treatment integrity of delivered psychotherapy was not assessed. Allegiance effect was even stronger where the experimenter was also both the developer of the preferred treatment and supervised or trained the therapists. No significant differences were found between allegiant and non-allegiant studies in terms of overall quality of studies.

**Conclusions:**

Experimenter’s allegiance influences the effect sizes of psychotherapy RCTs and can be considered non-financial conflict of interest introducing a form of optimism bias, especially since blinding is problematic in this kind of research. A clear reporting of EA in every single study should be given an opportunity to investigators of minimizing its overestimation effects.

**Electronic supplementary material:**

The online version of this article (doi:10.1186/s12991-015-0063-1) contains supplementary material, which is available to authorized users.

## Background

Allegiance in psychotherapy represents the therapist’s personal belief both in the superiority and the efficacy of a particular treatment [1–4]. Practicing therapists tend to choose a psychotherapy approach which is compatible with their beliefs and experience [2]. There is a hypothesis that the treatment effect can be larger when the therapist has proposed and developed the particular psychotherapy and/or supervised the therapist(s) applying it [4–6]. In psychotherapy research double-blind designs cannot be performed and therefore such experimenter biases are very difficult to be addressed [1–5, 7]. As a result, allegiance is a common factor existing across psychotherapy treatments that can influence the results of a conducted study [2–4].

The role of “therapeutic allegiance of the experimenter” [4] on psychotherapy research is a major concern and a long-standing debate has taken place [1, 2, 4, 5, 7]. Many investigators argue that experimenter’s allegiance (EA) can introduce systematic bias suggesting an adjustment in results of these studies in order to minimize the impact of allegiance [1, 8]. Sources of how EA could affect the outcome comprise poor training of therapists, the enthusiasm of the investigator for a particular treatment, the phenomenon of negative or null findings remain unpublished and the potential selection of biased therapists holding allegiances in crossed therapist study designs [1, 9]. However, others consider allegiance as a reflection of real differences between treatment comparisons (i.e., a better performance in treatment delivery of the preferred treatment) [1, 10, 11] recommending that adjustment for allegiance would lead to erroneous conclusions [10].

To date there is no sufficient evidence either to support or reject these arguments. Despite the fact that many researchers found that the outcomes of psychotherapies are influenced from allegiance to a specific school of thought [1, 4, 7, 12–14], the role of allegiance in the research field should be evaluated cautiously [1, 5, 15, 16]. Several meta-analyses have shown contradictory results between EA and treatment effect sizes in favor of the preferred treatment. For example, Miller et al. [17] in a meta-analysis regarding various childhood disorders found that the effect of allegiance on the variation of the true effects in the unconditional model reached 100 %. In contrast, other meta-analyses did not support that allegiance has an obvious and consolidated effect on the results of psychotherapeutic studies [15, 16]. Two further studies assessing EA as a primary outcome have also reported conflicting results [18, 19]. Briefly, Gaffan et al., found that the existing correlation among EA and treatment effect in cognitive therapy (CT) setting was not substantial [18]. Recently, Munder et al. advocated the hypothesis of bias resulting from methodological weaknesses in treatment comparisons [19].

In this work we aimed to systematically assess the potential influence of EA on a wide spectrum of psychotherapies and disorders comparing the magnitude of the effect sizes on allegiant vs. non-allegiant studies. To that end, we studied psychotherapy treatments included in meta-analyses of randomized controlled trials (RCTs) that have been published in Cochrane Library.

## Methods

### Search strategy and selection of reviews

We retrieved, from a previously published database that systematically assessed meta-analyses of RCTs for all types of psychotherapy and for various psychiatric and medical outcomes [20], all meta-analyses that included at least one RCT with clear evidence of EA. All studies were included in the Cochrane Database of Systematic Reviews (CDSR; Issue 9, 2010). We used only meta-analyses from the CDSR in order to eliminate the possibility of multiple recording of EA within meta-analyses on the same topic.

Specifically, we included meta-analyses with at least one study where one or more of the co-authors had developed the treatment or both developed the treatment and trained the therapists or both developed the treatment and supervised the therapists or supervised and/or trained the therapists alone. We included all studies where a certain type of psychotherapy treatment (authentic or not [21, 22]) for a wide range of medical and psychiatric disorders was directly compared with a non-experimental control arm (e.g., waiting list, usual care, standard care, no intervention and placebo). Meta-analyses that compare any psychotherapy treatment vs. medication or other alternative treatment as control group were also included. Meta-analyses with studies regarding study designs other than RCTs were excluded. Whenever a meta-analysis assessed the same outcome, we kept the version of the article that included the larger number of studies. No participant’s age restrictions were applied.

We did not include meta-analyses that examined a combination of psychotherapy and non-psychotherapy treatments (e.g., medication) if it was directly compared with another type of psychotherapy or meta-analyses evaluating direct comparisons between different types of psychotherapy. Meta-analyses assessing non-verbal techniques, web-based treatments and non-specific or miscellaneous treatments (e.g., yoga, dietary advice, recreation, biofeedback, etc.) were also excluded.

### Data extraction

From each eligible meta-analysis we recorded Cochrane ID, first author, publication year, diagnostic categories, number of analysis, control/comparison arms, number of RCTs of psychotherapy treatments included in meta-analysis, primary outcome investigated, type of data (continuous, dichotomous), the total number of participants, number of participants in comparison and control arm, the effect size (ES) of each study [standardized mean difference (SMD) or odds ratio (OR)] and its 95 % confidence intervals (CI). For each review, we extracted information on the main outcomes as they were reported by the Cochrane reviews. If the main outcome was not eligible, we recorded the secondary outcome that included the most studies in each meta-analysis. Whenever a systematic review was providing multiple comparisons, we recorded only the analysis that compared a psychotherapy with a non-experimental control arm. We followed the actual definition of each Cochrane review in order to categorize the type of psychotherapy treatments, i.e., cognitive behavioral therapy (CBT), psychoanalytic informed psychotherapies (PIPs), family systems therapy (FST), etc.

For each RCT included in the meta-analysis, we also recorded the existence of EA (Y/N), number of authors with allegiance, authentic approaches (also called bona fide treatment [21, 22] Y/N/Unclear), treatment integrity assessment (Y/N) and different levels of EA (e.g., developed the therapy or supervised or trained the therapists). We finally recorded the position of the “allegiant author” in the authorship list (first/last author vs. other position). In addition, data for risk of bias were extracted, according to the evaluation reported by the Cochrane reviews using the following domains of the risk of bias tool of the Cochrane Collaboration [23, 24]: methods for sequence generation and allocation concealment, blinding, and incomplete outcome data. Then for each single study the overall quality was classified as high (that is, low risk of bias for all domains), moderate (that is, unclear risk of bias for one or more domains in the absence of high risk of bias and low (that is, high risk of bias for one or more domains). Finally, we determined the quality of the delivered psychotherapy treatment according to the following criteria proposed by Furukawa et al. [25]: (A) high, when the psychotherapy alone arm was shown to be superior to the placebo arm in the same trial or when the same group of investigators had proved effectiveness in a separate randomized controlled trial; (B) moderate, when the conduct of psychotherapy was examined by a third reviewer through audiotapes, etc.; and (C) low, when the authors gave sufficiently detailed descriptions of the psychotherapy procedure.

One author (ED) examined the abstract of each of the identified references so as to evaluate the relevance of the meta-analytic studies. Whenever a study was eligible for inclusion, the full article was retrieved and two authors (ED, EE) extracted the data independently and they reached consensus in case of inconsistencies.

### Methods of rating the experimenter’s allegiance in included RCTs

Every single included RCT in eligible meta-analyses was independently reviewed by two investigators (ED, EE) for evidence of EA. The investigators reviewed the sections of introduction, method and reference list blind to results so as to assess whether one or more authors of the primary study were allegiant to the considered psychotherapy. We used a two-step process to code allegiance. We first rated evidence of allegiance in a continuum concept according to a six-point scale (from 0 to 5) proposed by Wampold et al. [26]. Then, we assigned an absolute allegiance rating of 1 (any level of EA = 1–5 in continuum concept) and 0 (no level of EA = 0 in continuum concept) for each psychotherapy treatment of the included RCTs [20]. We used the same distinction followed by Shadish and Montgomery [27] in order to create two groups of included RCTs (allegiant vs. non-allegiant) that would be feasible to be compared according to our study design.

### Analysis

To harmonize all the available information and allow for the synthesis of the evidence, we performed a transformation of the SMD to a logOR, whenever continuous data were available. This is based on the assumption that an underlying continuous variable produces a logistic distribution of equal standard deviation in the two intervention groups [28, 29].

From each comparison we obtained the summary OR for the allegiant studies (OR_A_) and the non-allegiant (OR_NA_) within the eligible meta-analyses using fixed effects models [30]. Then, we obtained a relative OR (ROR) from the comparison of OR_A_ vs. OR_NA_ for each eligible meta-analysis. A ROR which exceeds 1 equates to assessments that provide a more favorable response to the experimental treatment when a co-author is affiliated (e.g., developed the therapy, supervised or trained the therapists, etc.) to the psychotherapy under study compared to studies where non-experimenter’s allegiance is present.

To obtain a summary ROR across the meta-analyses, we combined the natural logarithm estimates of the RORs among all comparisons [31] using fixed and random effects [32]. Heterogeneity was evaluated with Cochran’s *Q* statistic (statistically significant for *P* < 0.10) and it was quantified with the *I*^2^ metric [33] (low, moderate, large, very large for values of <25, 25–49, 50–74, >75 %, respectively). When heterogeneity is absent (*I*^2^ = 0), random and fixed effects coincide.

All eligible comparisons were considered for the main analysis. A subgroup analysis was also performed according to the outcome (psychiatric disorders vs. other medical disorders), to the types of psychotherapy (e.g., CBT, PIPs, FST) and according to the type of data (binary vs. continuous). We also carried out sensitivity analyses limited in meta-analysis concerning bona fide treatments, treatment integrity, overall quality of study, overall quality of psychotherapy treatment, and ranking in the authorship list. We also performed a sensitivity analysis by level of allegiance. Specifically we created three groups where authors that have developed or trained or supervised the therapy correspond to the previously described rate of 5; the group where the author has developed the therapy corresponds to a rate of 4; and all other to rates 1–3. We have grouped the last category in order to have adequate power to analyze the results. Analyses were performed in STATA 10.0 (STATA Corp., College Station, TX, USA). *P* values are two tailed.

### Meta-regression

In order to examine further the potential sources of heterogeneity, a random effect meta-regression analysis was performed [34]. In this analysis we included the outcome, the type of psychotherapy, the type of the outcome (binary/continuous), the treatment integrity assessment, the overall quality of study and the overall quality of treatment delivery.

## Results

### Search results

From a previously published database that included 146 meta-analyses, we used the 60 systematic reviews from the CDSR [20]. These reviews were scrutinized further in depth for eligibility. Of these, we excluded 19 reviews because they did not meet our inclusion criteria and 41 were deemed eligible. In two out of 41 eligible meta-analyses, we were not able to clearly identify allegiance of any of the co-authors in the eligible RCTs and in 9 studies allegiance was present in all included RCTs and therefore they were excluded since we could not estimate a ROR for those meta-analyses. We finally included 30 reviews that met all the aforementioned inclusion criteria (see Additional file 1). The two independent researchers reached a very high level of agreement (216/240 studies) and in all cases (coding of allegiance or other disagreements in data extraction was discussed with a third researcher and a consensus was reached).

### Characteristics of eligible reviews

The thirty eligible meta-analyses (see Additional file 2) synthesized data from 240 RCTs, of which 18 (60 %) assessed binary and 12 (40 %) assessed continuous outcomes. Each meta-analysis included a median of 6 studies [interquantile range (IQR) 5–9] and a median of 4 studies with evidence of allegiance (IQR 3–5). The median number of participants per meta-analysis was 493 (IQR 239–803). The majority of meta-analyses (63.3 %) compared the efficacy or effectiveness of cognitive behavioral therapy versus control (*n* = 19). The outcome under study was various anxiety disorders (*n* = 6, 20.0 %), depression (*n* = 4, 13.3 %) and chronic pain (*n* = 4, 13.3 %). Sixteen meta-analyses assessed another outcome. Table 1 shows in detail the characteristics of included Cochrane reviews.

**Table 1:** Characteristics of eligible meta-analyses (m-a)

Characteristic; *n* (%), unless otherwise stated	Value
Number of eligible m-a	30 (100)
Number of included studies per m-a; median (IQR)	
Total	6 (5–9)
With ≥1 allegiance	4 (3–5)
With no allegiance	2 (1–4)
Sample size; median (IQR)	493 (239–803)
Meta-analysis favors	
Experimental arm	20 (66.7)
No significant difference	10 (33.3)
Type of data:	
Binary	18 (60.0)
Continuous	12 (40.0)
Types of psychotherapy:	
Cognitive behavior therapy (CBT)	19 (63.3)
Behavioral therapy (BT)	2 (6.7)
Family systems therapy (FST)	1 (3.3)
Psychological debriefing	1 (3.3)
Psychoanalytically informed psychotherapies (PIPs)	1 (3.3)
Supportive or counseling therapy	3 (10.5)
Variants of CBT	3 (10.5)
Outcomes:	
Anxiety disorders	6 (20.0)
Depression	4 (13.3)
Chronic pain	4 (13.3)
Eating disorders	2 (6.7)
Personality disorders	2 (6.7)
Substance use disorders	2 (6.7)
Smoking cessation	2 (6.7)
Asthma	1 (3.3)
Chronic fatigue syndrome	1 (3.3)
Common mental disorders	1 (3.3)
Mental illness and substance disorders	1 (3.3)
Schizophrenia	1 (3.3)
Tinnitus	1 (3.3)
Hypertension	1 (3.3)
Miscellaneous conditions (behavioral problems)	1 (3.3)

*IQR* interquartile range

### Comparison of RORs

**Fig. 1:**
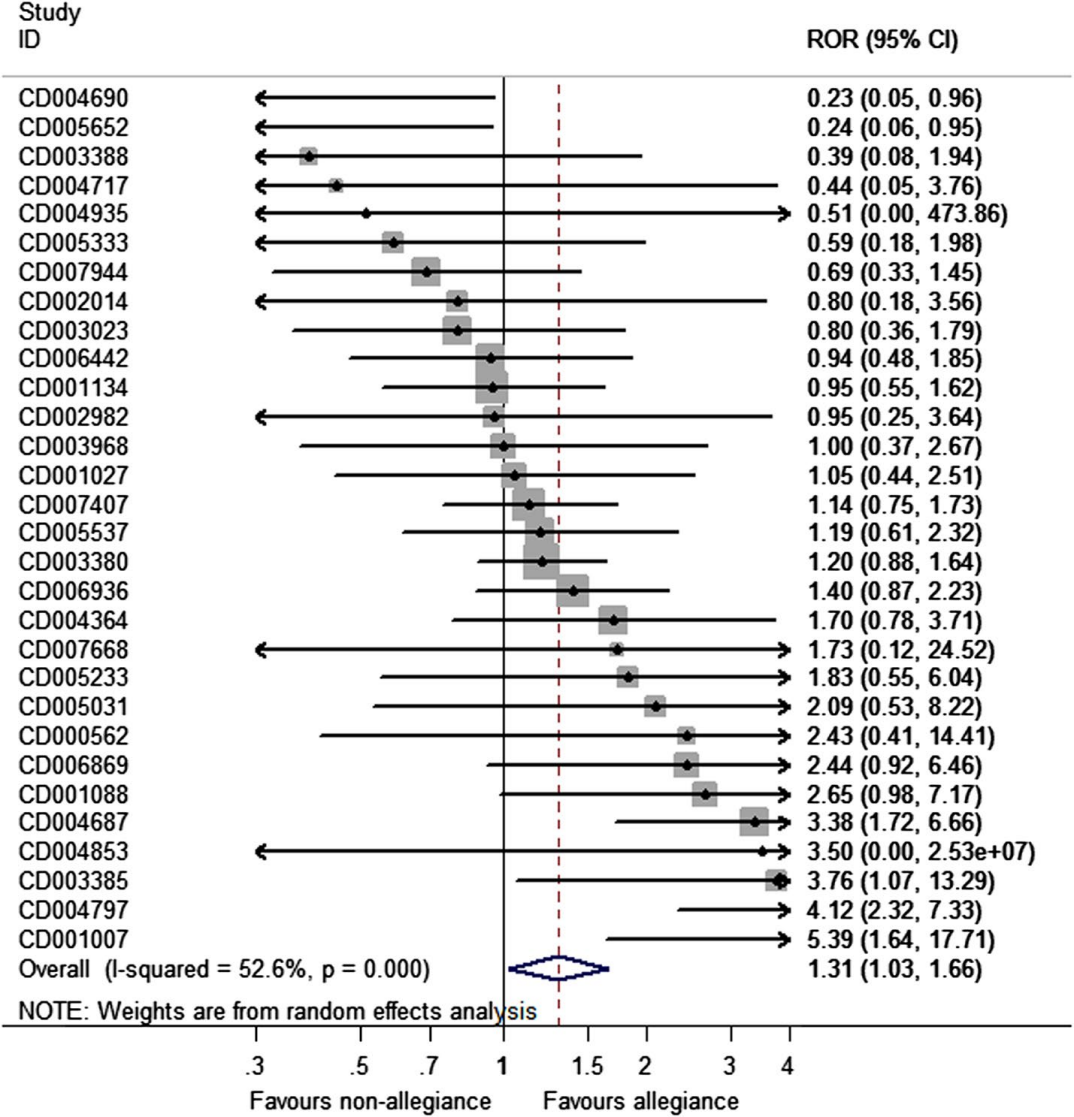
ROR and 95 % confidence intervals (CIs) for each comparison of an “allegiant” vs. “non-allegiant study”. The summary ROR has been calculated with random effects model. A ROR >1 favors allegiance; a ROR <1 favors non-allegiance

The summary ROR (sROR) was 1.31 (95 % CI 1.03–1.66, *P* = 0.030) using random effects model and 1.32 (95 % CI 1.14–1.52, *P* = 1.4 × 10^−4^ by fixed effects. Moderate heterogeneity was observed (*I*^2^ = 53 %) (Fig. 1). In 18 meta-analyses, the summary ROR was >1 showing that the experimental treatment was more favorable in studies where allegiance was present. Four out of those 18 studies were significant. In 12 meta-analyses the effect was in favor of non-allegiant studies and two out of those were significant.

Subgroup analyses using fixed and random effects are summarized in Table 2. Specifically, no significant difference was observed using random effects models regarding the outcome (psychiatric disorders vs. medical disorders) and type of data (binary vs. continuous). When we examined the effect of different types of psychotherapy treaments, we found that CBT had a sROR = 1.07 [(95 % CI 0.85–1.34), *P* = 0.580, *I*^*2*^ = 19 %]. For supportive or counseling therapy sROR = 1.44 [(95 % CI 1.01–2.05), *P* = 0.046, *I*^2^ = 0 %], whereas for other forms of psychotherapy sROR = 2.35 [(95 % CI 1.10–4.99), *P* = 0.027, *I*^2^ = 79 %].

**Table 2:** Summary RORs in various subgroup analyses

Characteristic	No of studies	Summary ROR (95 % CI) fixed effects	*P*	Summary ROR (95 % CI) random effects	*P*	*I* ^2^ (%)
Overall	30	1.32 (1.14–1.52)	1.4 × 10^−4^	1.31 (1.03–1.66)	0.030	53
Outcome						
Psychiatric disorders	20	1.37 (1.14–1.62)	5.4 × 10^−4^	1.30 (0.92–1.66)	0.143	64
Medical disorders	10	1.24 (0.97–1.57)	0.085	1.24 (0.97–1.84)	0.085	0
Types of psychotherapy						
CBT	22	1.10 (0.92–1.31)	0.311	1.07 (0.85–1.34)	0.580	19
Supportive or counseling	3	1.44 (1.01–2.05)	0.046	1.44 (1.01–2.05)	0.046	0
Other	5	2.22 (1.62–3.05)	8.7 × 10^−7^	2.35 (1.10–4.99)	0.027	79
Type of data						
Binary	18	1.50 (1.21–1.85)	2.2 × 10^−4^	1.39 (0.94–2.05)	0.097	21
Continuous	12	1.19 (0.99–1.44)	0.069	1.19 (0.93–1.51)	0.164	62

*ROR* relative odds ratio

### Sensitivity analysis

In a sensitivity analysis where we considered the position of the allegiance experimenter in the authorship list the sROR was 1.58 [(95 % CI 1.00–2.47) *P* = 0.048, *I*^2^ = 64 %] when the allegiant author was placed in another authorship position rather than the first or last and 1.25 [(95 % CI 1.00–1.56) *P* = 0.047, *I*^*2*^ = 35 %] when the author was first or last, using random effects model (Table 3). Regarding the various evidence of allegiance, the sROR was favorable for studies where the experimenter had developed the therapy with sROR = 1.36 [(95 % CI 1.07–1.72), *P* = 0.011, *I*^2^ = 49 %]. The magnitude of the effect was stronger in cases where the experimenter had both developed the therapy and supervised the therapists that conducted the treatment with sROR = 2.39 [(95 % CI 1.15–4.99), *P* = 0.020, *I*^2^ = 65 %], but it was attenuated or even disappeared in the other levels of EA such as training or supervision alone. In analysis of the bona fide treatments vs. non-bona fide vs. unclear bona fide treatments a significant allegiance effect in favor of non-bona fide treatments was found: sROR = 2.51 [(95 % CI 1.01–6.23), *P* = 0.048 and *I*^2^ = 88 %]. The analysis addressing the quality of studies did not show any significant association between allegiant and non-allegiant studies, whereas significant differences were observed in cases where treatment integrity was not evaluated (sROR = 1.54 [(95 % CI 1.01–2.35), *P* = 0.047 and *I*^*2*^ = 74 %]. Finally, the magnitude of the effect was stronger only in the case of low quality of the delivered psychotherapy sROR = 1.61 [(95 % CI 1.00–2.59), *P* = 0.049 and *I*^2^ = 76 %], as shown in Table 3.

**Table 3:** Summary RORs in sensitivity analysis for specific characteristics

Characteristic	*N*	Summary ROR (95 % CI) fixed effects	*P*	Summary ROR (95 % CI) random effects	*P*	*I* ^2^ (%)
Authorship list						
First/last position	30	1.26 (1.07–1.47)	0.005	1.25 (1.00–1.56)	0.047	35
Other position	20	1.53 (1.22–1.92)	2.5 × 10^−4^	1.58 (1.00–2.47)	0.048	64
Continuum level of allegiance						
Developed and supervised or trained (5)	6	2.22 (1.56–3.17)	1.2 × 10^−5^	2.39 (1.15–4.99)	0.020	65
Developed the therapy (4)	30	1.36 (1.18–1.57)	3.4 × 10^−5^	1.36 (1.07–1.72)	0.011	49
Other (1–3)	8	0.60 (0.38–0.96)	0.035	0.64 (0.31–1.30)	0.217	45
Bona fide treatment						
Yes	14	0.99 (0.79–1.24)	0.940	0.94 (0.68–1.29)	0.709	42
No	10	1.68 (1.27–2.22)	2.8 × 10^−4^	2.51 (1.01–6.23)	0.048	88
Unclear	4	1.97 (0.57–6.76)	0.280	1.06 (0.97–1.16)	0.173	0
Quality of studies						
High quality	15	1.28 (1.04–1.50)	0.021	1.54 (0.87–2.74)	0.141	78
Moderate quality	22	1.46 (1.12–1.89)	0.005	1.20 (0.73–1.99)	0.467	66
Low quality	7	0.41 (0.22–0.77)	0.041	0.53 (0.18–1.58)	0.257	54
Treatment integrity assessment						
Yes	9	1.05 (0.79–1.39)	0.763	0.99 (0.62–1.56)	0.952	53
No	28	1.29 (1.07–1.55)	0.007	1.54 (1.01–2.35)	0.047	74
Quality of psychotherapy						
High quality (A)	6	1.01 (0.66–1.55)	0.973	1.48 (0.91–2.42)	0.120	57
Unclear quality (B)	6	1.38 (0.84–2.28)	0.205	1.28 (0.49–3.30)	0.612	53
Low quality (C)	27	1.28 (1.05–1.56)	0.014	1.61 (1.00–2.59)	0.049	76

*N* number of studies that the comparison under study exists, *ROR* relative odds ratio

### Meta-regression

A meta-regression including the aforementioned variables revealed that only the assessment of treatment integrity was significantly contributing to the heterogeneity of sROR (regression coefficient = 0.56, *P* = 0.042) (see Additional file 3).

## Discussion

We evaluated RCTs of psychotherapy treatments included in 30 eligible meta-analyses and we have shown that there is an inflation of the reported effect when EA is present. Specifically, reported effect sizes were found to be larger by almost 30 % when the allegiant therapist had participated in the respective RCT compared to studies in which he was not included in the authorship list. Moderate heterogeneity was observed; however, this is well expected given the wide range of treatments and outcomes assessed in the analysis [35, 36].

To our knowledge, this is the first study that quantifies the observed effect and investigates the allegiance phenomenon in such a systematic manner across a spectrum of different psychotherapy treatments and outcomes. Other studies that tried to examine the experimenter allegiance hypothesis, focused only on specific psychotherapies or outcomes [5, 15–19, 26]. In our initial screening, we found that 1 out of 4 identified meta-analyses included only allegiant studies and from the 30 eligible meta-analyses almost 60 % of the primary studies included an investigator with evidence of allegiance as a co-author. It is evident that allegiance is present in a large number of RCTs of psychotherapy treatments, and therefore it should be carefully assessed and evaluated.

One may argue that the observed difference is not a biased inflation of the treatment effect, but could reflect the fact that the treatment offered by the therapist that developed the psychotherapy is simply superior to the treatment offered by other investigators [1, 10, 11]. However, we found that the inflation was not statistically significant when we limited our analysis in studies where authors have assessed the integrity of the delivered psychotherapy treatment. This could imply that EA may influence outcomes by affecting treatment adherence and competence [37]. This finding is consistent with the hypothesis that “therapist attitudes and openness” towards a particular treatment are likely to influence treatment integrity [38, 39], given that only the participants are blind to which treatment they receive [2, 5]. Therefore, it can be suggested that EA may act as systematic bias [2, 5, 19, 40] similar to optimism bias [41] rather than as a true efficacy effect [1, 10, 11] at least when the integrity of the psychotherapy is not evaluated. Optimism bias have been defined as an unwarranted belief in the efficacy of new treatments and a major overestimation factor that affecting RCTs [41]. This type of bias may be explained by the potential conflict between an experimenter’s allegiance to a school of thought and can be considered as a non-financial conflict of interest (COI) [42].

We also found that the EA effect is larger in supportive, counseling or other methods that include psychodynamic and family systems therapy. In contrast, even though an almost 10 % inflation was observed in CBT approach this effect was not significant. This could imply that CBT-related RCTs are well designed and the investigators have gained experience and methodological expertise due to training over the years, even though in our analysis there was no difference between the type of the psychotherapy and the quality of the study. Previous study by Gaffan et al. has argued that allegiance effect in CBT studies was more intense in earlier years but decreased over time [18]. Given that CBT is ranked at the highest level of empirical evidence compared to other types of psychotherapies in the literature [43], someone may argue that optimism bias can be observed in specific and new empirically supported approaches of psychotherapy. The latter is also supported by work of Luborsky et al. [5]. Of course, due to small number of comparisons, this result must be evaluated with caution.

Moreover, we found that the psychotherapy treatment effects are rather inflated when the experimenter both developed and supervised or trained the delivered therapy compared to other levels of allegiance. This finding is in accordance with other studies that found an association between level of allegiance and effect size [17]. One possible explanation is that the developers of specific psychotherapy treatments show more interest for the evidence-based practice of their own therapies compared to others [42]. Along with the fact that investigators’ psychotherapy preferences tend to produce more positive results [44] experimenter’s allegiance effect may inflate the relative efficacy of various psychotherapies in a familiar manner that COI impacts on the relative efficacy of the various drug trials [42]. COI(s) have been defined as a set of conditions in which professional judgment concerning a primary interest (e.g., patient welfare, research validity) may be unduly influenced by a secondary interest (e.g., personal recognition, career advancement or visibility in the media; bestowing favor on a relative, friend or colleague; the allegiance to a school of thought) [45]. Many investigators have found that industry sponsorship trials are more likely to have favorable outcomes (e.g., efficacy or safety) than independently financed drug trial [46–49]. It has been shown that odds of positive results are more that twofold larger when COI exists, which is comparable with our findings here [48].

We also found a significant inflation in non-bona fide treatments (non-authentic) in contrast to bona fide (authentic) and unclear bona fide treatments. This finding indicates that absence of investigators treatment integrity may allow allegiance to interfere, therefore it could be considered as one of the potential sources of allegiance bias [37]. Moreover, low quality of the delivered psychotherapy may also include a plausible rationale for the allegiance effect. Other factors such as the ranking of the investigators in the authorship list and the quality of the study design do not seem to significantly contribute to this effect.

There are some caveats to the findings of this meta-epidemiological study. First, we did not explore the potential interaction of the authors of the non-allegiant studies with the “allegiant therapist”. It is possible that some authors or even the leading author could be a former trainee, a student or even a colleague of the “allegiant” scientist. Therefore, he might be equally well trained for the application of the specific psychotherapy treatment. However, it is expected that this would lead to an underestimation of our effects rather than to the observed inflation. Similarly, we did not explore the possibility that more than one author were coded as EA. We have inadequate power perform such an analysis, due the very low number of comparisons given that <6 and <1 % of the studies had two or three allegiant authors, respectively. Finally, we based our evaluation only on the limited information available in the published articles and therefore may we have not identified all allegiant studies. However, other studies that have identified links between allegiance and psychotherapeutic studies results have similarly relied upon identical sources and types to measure allegiance. [15–19, 26, 27]

## Conclusions

In this study, we show that experimenter’s allegiance effect inflates the reported effect sizes in randomized controlled trials in psychotherapy by 30 %. This treatment effect could be attributed to different components such as the superiority of the treatment given by an experienced therapist that proposed the intervention. However, a distinct component could arise due to systematic biases and affect the reported effects in a way similar to conflict of interest observed in medical research literature. Therefore, even though this is a non-financial conflict of interest, we strongly suggest that experimenter’s allegiance should be clearly reported in every single randomized controlled trial and meta-analysis of psychotherapeutic treatments. This becomes apparent as our group has shown that allegiance is seldom reported in RCTs and meta-analyses [20]. Adequate documentation will allow for the assessment of the possible allegiance in each study and will also provide the opportunity to the investigators to control for allegiance using previously proposed techniques [1, 2, 5, 12, 13, 17–19, 50].

## Additional files

**Additional file 1.** Supplementary references of Cochrane reviews. Additional file 2:
**Table S1.** Eligible meta-analyses. Additional file 3:
**Table S2.** Results from meta-regression analysis.

## Abbreviations

CBT: cognitive behavioral therapy
CDSR: Cochrane Database of Systematic Reviews
CI: confidence intervals
COI(s): conflicts of interest
CT: cognitive therapy
EA: experimenter’s allegiance
ES: effect size
FST: family systems therapy
IQR: interquartile range
OR: odds ratio
OR_A_: odds ratio for allegiant studies
OR_NA_: odds ratio for non-allegiant studies
PIPs: psychoanalytic informed psychotherapies
RCTs: randomized controlled trials
ROR: relative odds ratio
SMD: standardized mean difference
sROR: summary relative odds ratio
