# Supplementary material for: A systematic appraisal of allegiance effect in randomized controlled trials of psychotherapy
Source: Ann Gen Psychiatry. 2015 Sep 15;14:25. doi: 10.1186/s12991-015-0063-1 (PMC4570291; doi:10.1186/s12991-015-0063-1)
Supplement: Supplementary file 1 — Additional file 1. Supplementary references of Cochrane reviews. [file 12991_2015_63_MOESM1_ESM.docx]

**Supplementary References of Cochrane reviews**

1. Martinez-Devesa P, Perera R, Theodoulou M, Waddell A. Cognitive behavioural therapy for tinnitus. *Cochrane Database Syst Rev*. 2010 Sep 8; (9):CD005233.

2. Gibbon S, Duggan C, Stoffers J, Huband N, Völlm BA, Ferriter M, Lieb K. Psychological interventions for antisocial personality disorder. Cochrane *Database Syst Rev.* 2010 Jun 16; (6):CD007668.

3. Lai DT, Cahill K, Qin Y, Tang JL. Motivational interviewing for smoking cessation. *Cochrane Database Syst Rev.* 2010 Jan 20; (1):CD006936.

4. Akechi T, Okuyama T, Onishi J, Morita T, Furukawa TA. Psychotherapy for depression among incurable cancer patients. *Cochrane Database Syst Rev*. 2008 Apr 16;(2):CD005537.

5. Henschke N, Ostelo RW, van Tulder MW, Vlaeyen JW, Morley S, Assendelft WJ, Main CJ. Behavioural treatment for chronic low-back pain. *Cochrane Database Syst Rev.* 2010 Jul 7; (7):CD002014.

6. Roberts NP, Kitchiner NJ, Kenardy J, Bisson J. Multiple session early psychological interventions for the prevention of post-traumatic stress disorder. *Cochrane Database Syst Rev*. 2009 Jul 8; (3):CD006869.

7. Eccleston C, Williams AC, Morley S. Psychological therapies for the management of chronic pain (excluding headache) in adults. *Cochrane Database Syst Rev*. 2009 Apr 15; (2):CD007407.

8. Eccleston C, Palermo TM, Williams AC, Lewandowski A, Morley S. Psychological therapies for the management of chronic and recurrent pain in children and adolescents. *Cochrane Database Syst Rev*. 2009 Apr 15; (2):CD003968.

9. Zijdenbos IL, de Wit NJ, van der Heijden GJ, Rubin G, Quartero AO. Psychological treatments for the management of irritable bowel syndrome. *Cochrane Database Syst Rev*. 2009 Jan 21; (1):CD006442.

10. Price JR, Mitchell E, Tidy E, Hunot V. Cognitive behaviour therapy for chronic fatigue syndrome in adults. *Cochrane Database Syst Rev*. 2008 Jul 16; (3):CD001027.

11. Gava I, Barbui C, Aguglia E, Carlino D, Churchill R, De Vanna M, McGuire HF. Psychological treatments versus treatment as usual for obsessive compulsive disorder (OCD). *Cochrane Database Syst Rev*. 2007 Apr 18; (2):CD005333.

12. Abbass AA, Hancock JT, Henderson J, Kisely S. Short-term psychodynamic psychotherapies for common mental disorders. *Cochrane Database Syst Rev*. 2006 Oct 18; (4):CD004687.

13. Yorke J, Fleming SL, Shuldham CM. Psychological interventions for adults with asthma. *Cochrane Database Syst Rev*. 2006 Jan 25;(1):CD002982.

14. James A, Soler A, Weatherall R. Cognitive behavioural therapy for anxiety disorders in children and adolescents. *Cochrane Database Syst Rev*. 2005 Oct 19; (4):CD004690.

15. Crawford-Walker CJ, King A, Chan S. Distraction techniques for schizophrenia. *Cochrane Database Syst Rev*. 2005 Jan 25;(1):CD004717.

16. Dennis CL, Creedy D. Psychosocial and psychological interventions for preventing postpartum depression. *Cochrane Database Syst Rev.* 2004 Oct 18; (4):CD001134.

17. Merry S, McDowell H, Hetrick S, Bir J, Muller N. Psychological and/or educational interventions for the prevention of depression in children and adolescents. *Cochrane Database Syst Rev.* 2004; (1):CD003380.

18. Roberts NP, Kitchiner NJ, Kenardy J, Bisson JI. Early psychological interventions to treat acute traumatic stress symptoms. *Cochrane Database Syst Rev*. 2010 Mar 17; (3):CD007944.

19. Hay PP, Bacaltchuk J, Stefano S, Kashyap P. Psychological treatments for bulimia nervosa and binging. *Cochrane Database Syst Rev.* 2009 Oct 7; (4):CD000562.

20. Wilson KC, Mottram PG, Vassilas CA. Psychotherapeutic treatments for older depressed people. *Cochrane Database Syst Rev*. 2008 Jan 23; (1):CD004853.

21. Furukawa TA, Watanabe N, Churchill R. Combined psychotherapy plus antidepressants for panic disorder with or without agoraphobia. Cochrane *Database Syst Rev*. 2007 Jan 24; (1):CD004364.

22. Binks CA, Fenton M, McCarthy L, Lee T, Adams CE, Duggan C. Psychological therapies for people with borderline personality disorder. *Cochrane Database Syst Rev.* 2006 Jan 25; (1):CD005652.

23. Littell JH, Popa M, Forsythe B. Multisystemic Therapy for social, emotional, and behavioral problems in youth aged 10-17. *Cochrane Database Syst Rev*. 2005 Oct 19; (4):CD004797.

24. Bacaltchuk J, Hay P, Trefiglio R. Antidepressants versus psychological treatments and their combination for bulimia nervosa. *Cochrane Database Syst Rev.* 2001; (4):CD003385.

25. Bisson J, Andrew M. Psychological treatment of post-traumatic stress disorder (PTSD). *Cochrane Database Syst Rev*. 2007 Jul 18; (3):CD003388.

26. Amato L, Minozzi S, Davoli M, Vecchi S, Ferri MM, Mayet S. Psychosocial and pharmacological treatments versus pharmacological treatments for opioid detoxification. *Cochrane Database Syst Rev*. 2008 Oct 8; (4):CD005031.

27. Dickinson HO, Campbell F, Beyer FR, Nicolson DJ, Cook JV, Ford GA, Mason JM. Relaxation therapies for the management of primary hypertension in adults. *Cochrane Database Syst Rev.*2008 Jan 23; (1):CD004935.

28. Cleary M, Hunt G, Matheson S, Siegfried N, Walter G. Psychosocial interventions for people with both severe mental illness and substance misuse. Cochrane *Database Syst Rev*. 2008 Jan 23; (1):CD001088.

29. Knapp WP, Soares BG, Farrel M, Lima MS. Psychosocial interventions for cocaine and psychostimulant amphetamines related disorders. *Cochrane Database Syst Rev.* 2007 Jul 18; (3):CD003023.

30. Stead LF, Lancaster T. Group behaviour therapy programmes for smoking cessation. *Cochrane Database Syst Rev*. 2005 Apr 18; (2):CD001007.
