# Supplementary material for: A systematic appraisal of allegiance effect in randomized controlled trials of psychotherapy
Source: Ann Gen Psychiatry. 2015 Sep 15;14:25. doi: 10.1186/s12991-015-0063-1 (PMC4570291; doi:10.1186/s12991-015-0063-1)
Supplement: Additional file 2: — Table S1. Eligible meta-analyses. [file 12991_2015_63_MOESM2_ESM.docx]

**Table S1: Eligible meta-analyses**

| **Supl.Ref^*^** | **Cochrane ID** | **Outcome** | **Experimental Intervention** | **Control Arm** | **T_n_** | **A_n_** | **TSS_n_** |
| --- | --- | --- | --- | --- | --- | --- | --- |
| 1 | CD005233 | Tinnitus | CBT | WL | 6 | 5 | 354 |
| 2 | CD007668 | Antisocial personality disorder | CM plus standard care | standard care | 2 | 1 | 127 |
| 3 | CD006936 | Smoking cessation | MI | TAU | 14 | 4 | 10553 |
| 4 | CD005537 | Depression in cancer patients | Supportive psychotherapy/CBT / problem -solving | TAU | 6 | 4 | 517 |
| 5 | CD002014 | Chronic pain | CBT | WL | 5 | 4 | 239 |
| 6 | CD006869 | Post traumatic stress disorder | Counseling / Debriefing | WL/TAU | 5 | 3 | 479 |
| 7 | CD007407 | Chronic pain | CBT | TAU | 23 | 10 | 1199 |
| 8 | CD003968 | Chronic pain | CBT/BT | W/L/TAU/no treatment/pharmacological treatment | 15 | 10 | 589 |
| 9 | CD006442 | Irritable bowel syndrome | CBT/BT /IPT | W/L/TAU/no treatment/pharmacological treatment | 7 | 5 | 492 |
| 10 | CD001027 | Chronic fatigue syndrome | CBT | TAU | 4 | 2 | 307 |
| 11 | CD005333 | Obsessive compulsive disorder | DIRT /CBT/BT | TAU | 9 | 3 | 230 |
| 12 | CD004687 | Common mental disorders | Short psychodynamic therapy | WL/TAU | 10 | 6 | 602 |
| 13 | CD002982 | Asthma | BT/hypnosis | WL/TAU | 5 | 1 | 249 |
| 14 | CD004690 | Anxiety disorders | CBT | TAU/WL/advice | 12 | 10 | 760 |
| 15 | CD004717 | Schizophrenia | Distraction therapy | Health promotion+ standard care | 4 | 2 | 124 |
| 16 | CD001134 | Postpartum depression | Debriefing/IPT | TAU | 6 | 1 | 2983 |
| 17 | CD003380 | Depression | CBT/ problem -solving | TAU | 9 | 6 | 2270 |
| 18 | CD007944 | Acute traumatic stress symptoms | TFCBT | WL | 5 | 4 | 428 |
| 19 | CD000562 | Bulimia nervosa | CBT | WL | 6 | 5 | 243 |
|  |  |  |  |  |  |  |  |
| "(cont)."  20 | CD004853 | Depression in older people | CBT | WL | 5 | 4 | 141 |
| 21 | CD004364 | Panic disorder+/-agoraphobia | CBT/BT /Short psychodynamic therapy | Pharmacological treatment | 9 | 3 | 543 |
| 22 | CD005652 | Borderline personality disorder | Dialectical behavior therapy | TAU | 3 | 1 | 155 |
| 23 | CD004797 | Behavioral problems in youth | MST | TAU | 5 | 4 | 803 |
| 24 | CD003385 | Bulimia nervosa | Antidepressants | CBT | 5 | 4 | 237 |
| 25 | CD003388 | Post traumatic stress disorder | TFCBT/CBT/Prolonged exposure/EMDR | WL/TAU | 14 | 11 | 716 |
| 26 | CD005031 | Opioid detoxification | CM+pharmacological treatment | Pharmacological treatment | 5 | 2 | 184 |
| 27 | CD004935 | Hypertension | CBT/BT | Placebo | 21 | 9 | 1049 |
| 28 | CD001088 | Severe mental illness and substance misuse | CBT/ MI | TAU | 8 | 5 | 493 |
| 29 | CD003023 | Psychostimulant use disorder | CBT | Drug Counseling | 6 | 5 | 961 |
| 30 | CD001007 | Smoking cessation | BT | No intervention | 7 | 4 | 965 |

* = All eligible meta-analyses were analyzed randomized controlled trials (RCTs) only and had at least one experimenter’s allegiance (EA) study.

CBT= Cognitive Behavioral Therapy; MI= Motivational Interviewing; BT= Behavioral Therapy; CM= Contingency Management; IPT= Interpersonal psychotherapy; DIRT=Danger Ideation Reduction Therapy; TFCBT= Trauma Focused Cognitive Behavioral Therapy; MST= Multisystemic Therapy; EMDR=Eye movement desensitization and reprocessing

WL=Waiting list; TAU= Treatment as usual

T_n_ = number of trials

A_n_ = number of trials with allegiance (A<T)

TSS_n_ = Total Sample Size (number of participants)
