# Supplementary material for: A systematic appraisal of allegiance effect in randomized controlled trials of psychotherapy
Source: Ann Gen Psychiatry. 2015 Sep 15;14:25. doi: 10.1186/s12991-015-0063-1 (PMC4570291; doi:10.1186/s12991-015-0063-1)
Supplement: Additional file 3: — Table S2. Results from meta-regression analysis. [file 12991_2015_63_MOESM3_ESM.docx]

**Table S2: Results from meta-regression analysis**

| **Variable** | | **B** | **SE** | ***P*** |  |  |
| --- | --- | --- | --- | --- | --- | --- |
| Model | |  |  |  |  |  |
| - Condition | | | -.035 | .261 | .894 |  |
| - Psychotherapy Type | .319 | .169 | .072 |  |  |  |
| - Outcome | -.234 | .265 | .386 |  |  |  |
| - Integrity Assessment* | .560 | .260 | .042 |  |  |  |
| - Overall Quality of Psychotherapy* | -.184 | .248 | .464 |  |  |  |
| - Overall Quality of Studies* | .028 | .175 | .872 |  |  |  |

Coding of variables: Condition group (Medical=1, Mental=2),

Psychotherapy Type group (Cognitive Behavioral Therapy=1,

Supportive or Counseling=2, Other =3), Outcome group

(Continuum=1, Binary=0), Integrity Assessment (Yes=2, No=1),

Overall Quality of Psychotherapy (High (A) =3,

Unclear (B) =2, Low(C)=1), Overall quality of studies

(High=3, Moderate=2, Low=1).

* We used the median value from allegiant studies vs. non allegiant studies
